# Supplementary material for: The Effectiveness of Heat-Killed Pediococcus acidilactici K15 in Preventing Respiratory Tract Infections in Preterm Infants: A Pilot Double-Blind, Randomized, Placebo-Controlled Study
Source: Nutrients. 2024 Oct 25;16(21):3635. doi: 10.3390/nu16213635 (PMC11547538; doi:10.3390/nu16213635)
Supplement: Supplementary file 1 [file nutrients-16-03635-s001.zip › nutrients-3228422-supplementary.pdf]

Figure S1. Comparison of alpha diversity before and after the study period in both groups described by rarefaction curves of Chao1

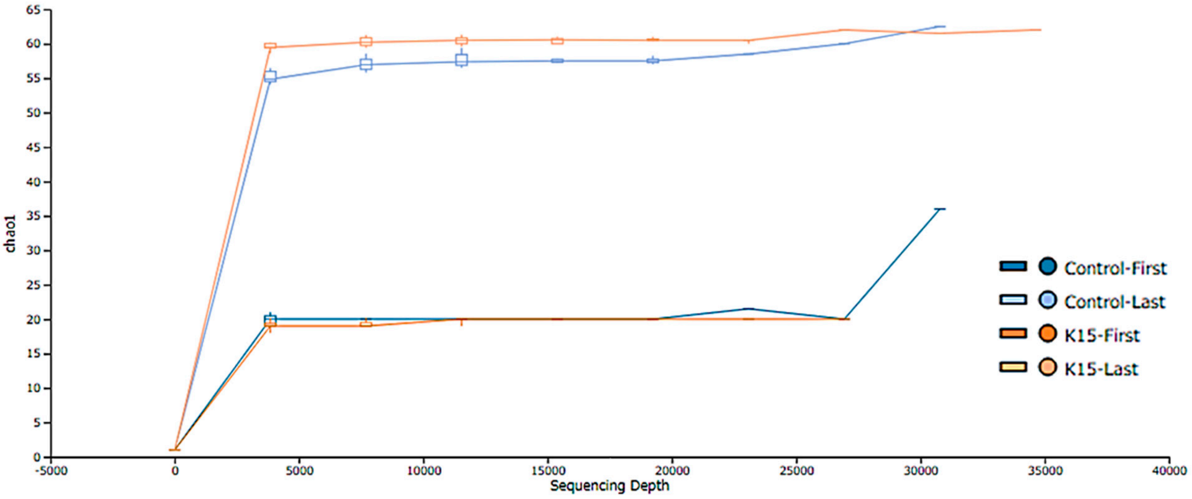

Figure S2. Comparison of beta diversity before and after the study period in both groups

Figure S2a. Weighted UniFrac distances by Principal Coordinate Analysis

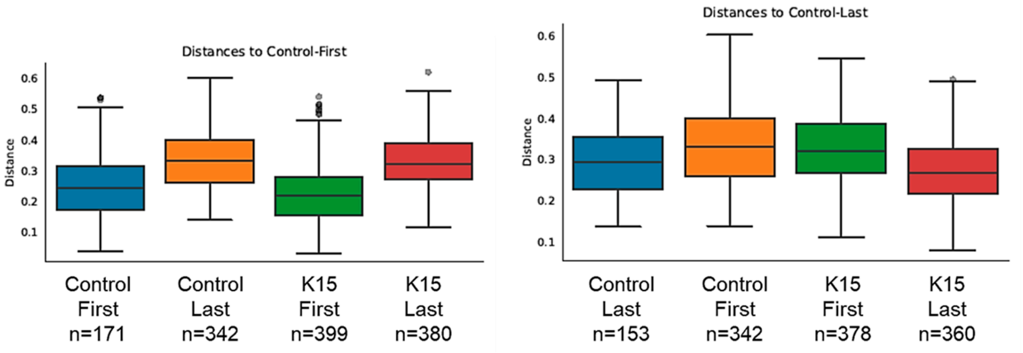

Figure S2b. PERMANOVA analysis

Group 1	Group 2	Sample size	Permutations	pseudo-F	p-value	q-value
Control-First	Control-Last	37	999	8.960187	0.001	0.0015
Control-First	K15-First	40	999	0.49922	0.735	0.735
Control-Last	K15-Last	38	999	1.047476	0.364	0.4368
K15-First	K15-Last	41	999	20.72793	0.001	0.0015

※Control-First: n=19, Control-Last: n=18, K15-First: n=21, K15-Last: n=20

Table S1. The mean number of febrile days (≥37.5℃) (Subgroup analysis) in the K15 and placebo group

Subgroup		N	Mean	SD	Difference (K15 - Placebo)			
					Mean	SD	95% CI	p-value
High adherence rate	K15	10	3.7	4.2	-1.8	4.7	[-6.0, 2.5]	0.3369
	Placebo	11	5.5	5.1				
Low frequency of LAB intake	K15	13	4.7	7.8	-2.5	7.1	[-9.8, 4.9]	0.101
	Placebo	6	7.2	4.9				
Subjects with older siblings	K15	10	2.4	2.4	-5.7	6.8	[-12.6, 1.1]	0.0482
	Placebo	8	8.1	9.9				
Nursery school attendance at one year old	K15	6	3.8	5.3	-6.5	9	[-18.0, 5.0]	0.146
	Placebo	6	10.3	11.5				

CI: confidence interval; LAB: lactic acid bacterium.

Table S2. The antibody titers against Hepatitis B at the end of the study periods, µg/mL (mean±SD)

K15 (n=19*)	Placebo (n=18)	*p*-value
2113±4783	1849±2143	0.8185

*One infant could not be collected blood sample
